# PI3K-Akt-mTOR inhibition by GNE-477 inhibits renal cell carcinoma cell growth *in vitro* and *in vivo*

**DOI:** 10.18632/aging.103221

**Published:** 2020-05-18

**Authors:** Xueting Ye, Jian-Wei Ruan, Hang Huang, Wei-Ping Huang, Yan Zhang, Fangyi Zhang

**Affiliations:** 1 Department of Urology, The First Affiliated Hospital of Wenzhou Medical University, Wenzhou, China; 2 Department of Orthopedics, Taizhou Municipal Hospital, Taizhou, China; 3 Department of Radiotherapy and Oncology, Affiliated Kunshan Hospital of Jiangsu University, Kunshan, China

**Keywords:** renal cell carcinoma, PI3K-Akt-mTOR, GNE-477, molecularly-targeted therapies

## Abstract

Sustained activation of PI3K-Akt-mTOR cascade is important for renal cell carcinoma (RCC) cell progression. GNE-477 is a novel and efficacious PI3K-mTOR dual inhibitor. The current study tested its anti-RCC cell activity. In the primary cultured human RCC cells, GNE-477 potently inhibited cell growth, viability and proliferation, as well as cell cycle progression, migration and invasion. Furthermore, it induced robust apoptosis activation in primary RCC cells, but being non-cytotoxic to HK-2 epithelial cells and primary human renal epithelial cells. In the primary RCC cells GNE-477 inactivated PI3K-Akt-mTOR cascade by blocking phosphorylation of p85, Akt1, p70S6K1 and S6. Restoring Akt-mTOR activation by a constitutively-active Akt1 reversed GNE-477-induced anti-RCC cell activity. In nude mice intraperitoneal injection of GNE-477 potently suppressed RCC xenograft tumor growth. Collectively, targeting PI3K-Akt-mTOR cascade by GNE-477 inhibits RCC cell growth *in vitro* and *in vivo*.

## INTRODUCTION

Renal cell carcinoma (RCC) is a common malignancy in the World [1–4], causing a large number of cancer-associated human mortalities annually [5, 6]. Significantly, its incidence has been rising in recent years [5, 6]. Currently, a large proportion of human RCC patients are diagnosed at late- and advanced-stages with poor prognosis [1–4]. It is therefore essential to explore novel oncogenes or therapeutic targets of RCC [1–4].

Molecularly-targeted therapies are currently needed for better and advanced RCC treatments [7]. Our previous studies have shown that melanoma antigen A6 (MAGEA6), a cancer-specific ubiquitin ligase of AMP-activated protein kinase (AMPK), is uniquely expressed in human RCC tissues and cells. MAGEA6 silencing or knockout activated AMPK signaling to inhibit mammalian target of rapamycin (mTOR) cascade, thereby inhibiting RCC cell progression [8]. Furthermore, a long non-coding RNA (LncRNA) THOR is expressed in RCC tissues and cells. THOR silencing resulted in potent RCC cell growth inhibition *in vitro* and *in vivo* [9].

In RCCs, several mutations, including the activating mutations of PIK3CA, depletion or loss-of-function mutations of PTEN, or constitutive activation of multiple receptor tyrosine kinases, are commonly detected. These mutations will lead to profound and sustained PI3K-Akt-mTOR cascade activation, associated with RCC progression and therapy resistance [10–13]. Overactivation of PI3K-Akt-mTOR signaling is vital for RCC cell proliferation, survival, migration and metastasis, as well as angiogenesis and treatment resistance [10–13]. Conversely, pharmacological inhibitors of this cascade have displayed promising and important therapeutic values for RCC [10–13]. Several mTOR-inhibitors, including temsirolimus and everolimus, are currently being utilized for the treatment of certain RCCs [10–13].

A very recent study by Heffron et al., has identified GNE-477 as a potent and efficient PI3K and mTOR dual inhibitor [14]. By simultaneously targeting PI3K and mTOR, GNE-477 may have unique advantage over single-specific mTORC1 or PI3K inhibitors in inhibiting human cancer cells [14]. The results of this study will show that targeting PI3K-Akt-mTOR cascade by GNE-477 potently inhibits RCC cell growth *in vitro* and *in vivo*.

## RESULTS

### GNE-477 potently inhibits human RCC cell survival, proliferation, cell cycle progression, migration and invasion

**Figure 1:**
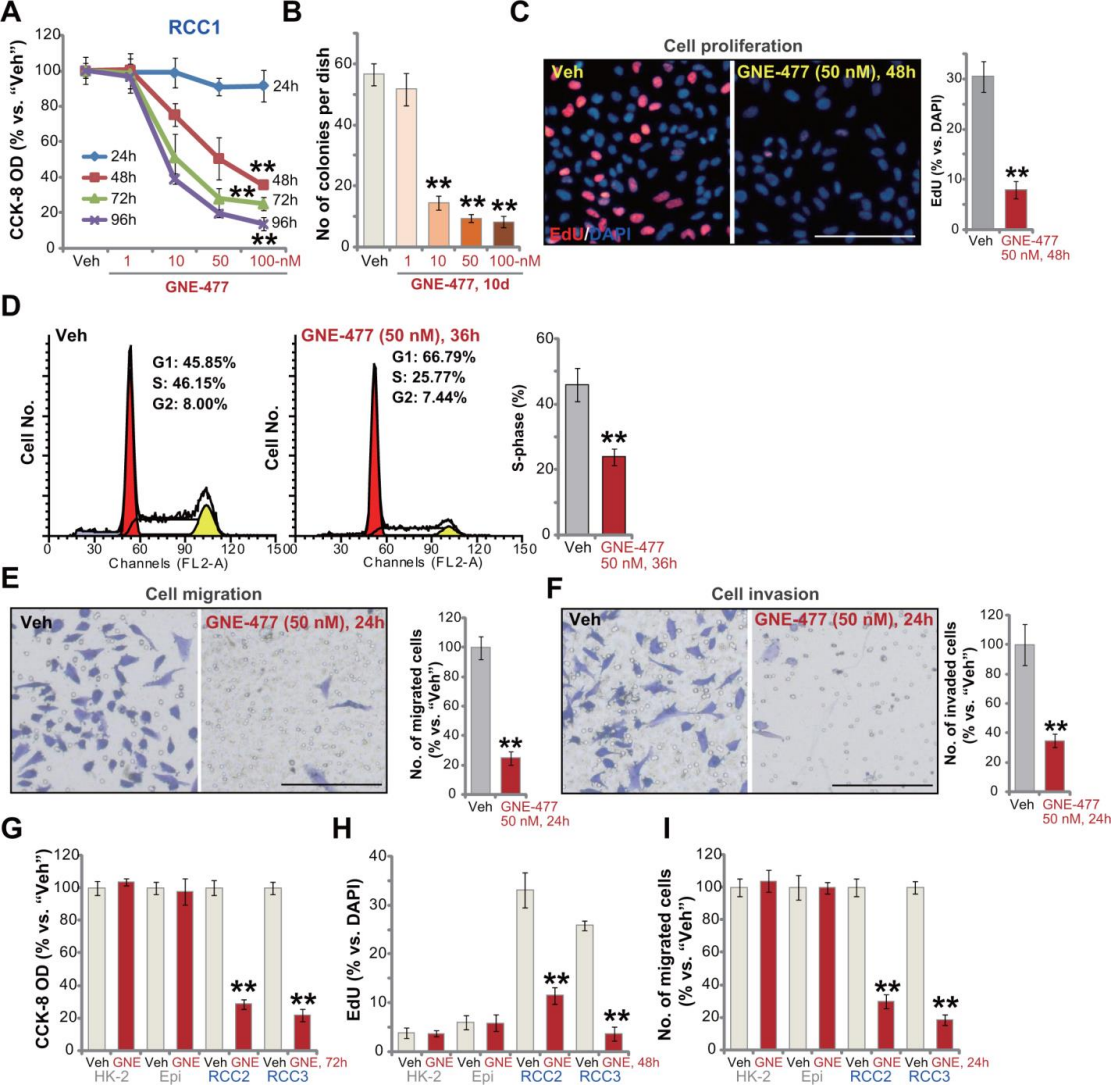
**GNE-477 potently inhibits human RCC cell progression *in vitro*.** The primary human RCC cells (“RCC1/RCC2/RCC3”), HK-2 renal epithelial cells (“HK-2”) or the primary human renal epithelial cells (“Epi”) were treated with applied concentration of GNE-477 (1-100 nM) or the vehicle control (“Veh”, 0.1% DMSO), cells were further cultured for the designated time periods (24-96h); Cell viability (**A**, **G**), colony formation (**B**) and proliferation (**C**, **H**) as well as cell cycle progression (**D**), migration (**E**, **I**) and invasion (**F**) were tested by the assays mentioned in the text. Bars stand for mean ± standard deviation (S.D.). For each assay, n=5. ** *p* < 0.01 *vs.* “Veh” cells. Experiments in this figure were repeated five times, and similar results obtained. Scale bar= 100 μm (**C**, **E**, **F**).

First the primary human RCC cells (“RCC1” [8, 9]) were cultured in FBS-containing complete medium, and treated with GNE-477 at 1-100 nM. After further culture for 24-96h, the cell viability was tested by CCK-8 assays. As demonstrated, GNE-477, in a dose-dependent manner, efficiently decreased CCK-8 viability in RCC1 cells (Figure 1A). The dual PI3K-mTOR inhibitor also displayed a time-dependent response in inhibiting CCK-8 viability in RCC1 cells (Figure 1A). The CCK-8 OD reduction was significant at 48h after GNE-477 treatment (10-100 nM), that lasted for at least 96h (Figure 1A). The colony formation assay results, Figure 1B, show that the number of viable RCC1 colonies was significantly decreased following GNE-477 treatment (at 10-100 nM, for 10 days). Since in RCC1 cells 50 nM of GNE-477 resulted in potent cell viability reduction (Figure 1A) and colony formation inhibition (Figure 1B), this concentration was selected for further experiments.

To study cell proliferation, a nuclear EdU staining assay was performed. Results show that GNE-477 (50 nM, 48h) treatment robustly inhibited EdU incorporation (EdU/DAPI%) in RCC1 cells (Figure 1C). Analyzing cell cycle progression by FACS, we show that S phases were potently decreased in GNE-477-treated RCC1 cells (Figure 1D), where G1 phases were increased (Figure 1D). Further studies demonstrated that GNE-477 (50 nM, 24h) suppressed *in vitro* cell migration (Figure 1E) and invasion (Figure 1F), tested by “Transwell” (Figure 1E) and “Matrigel Transwell” (Figure 1F) assays, respectively. Notably, for cell migration/invasion assays, RCC1 cells were treated with GNE-477 (50 nM) for only 24h, when no significant viability reduction was detected (Figure 1A).

In the primary human RCC cells-derived from two other RCC patients, RCC2 and RCC3, GNE-477 (50 nM) stimulation potently inhibited cell viability (CCK-8 OD, Figure 1G), proliferation (nuclear EdU incorporation, Figure 1H) and migration (Figure 1I). In contrast, in HK-2 renal epithelial cells and primary human renal epithelial cells, the same GNE-477 (50 nM) treatment was completely ineffective and non-cytotoxic (Figure 1G–1I). These results show that GNE-477 specially and potently inhibited RCC cell viability, proliferation, cell cycle progression, migration and invasion *in vitro*.

### GNE-477 induces apoptosis activation in primary human RCC cells

**Figure 2:**
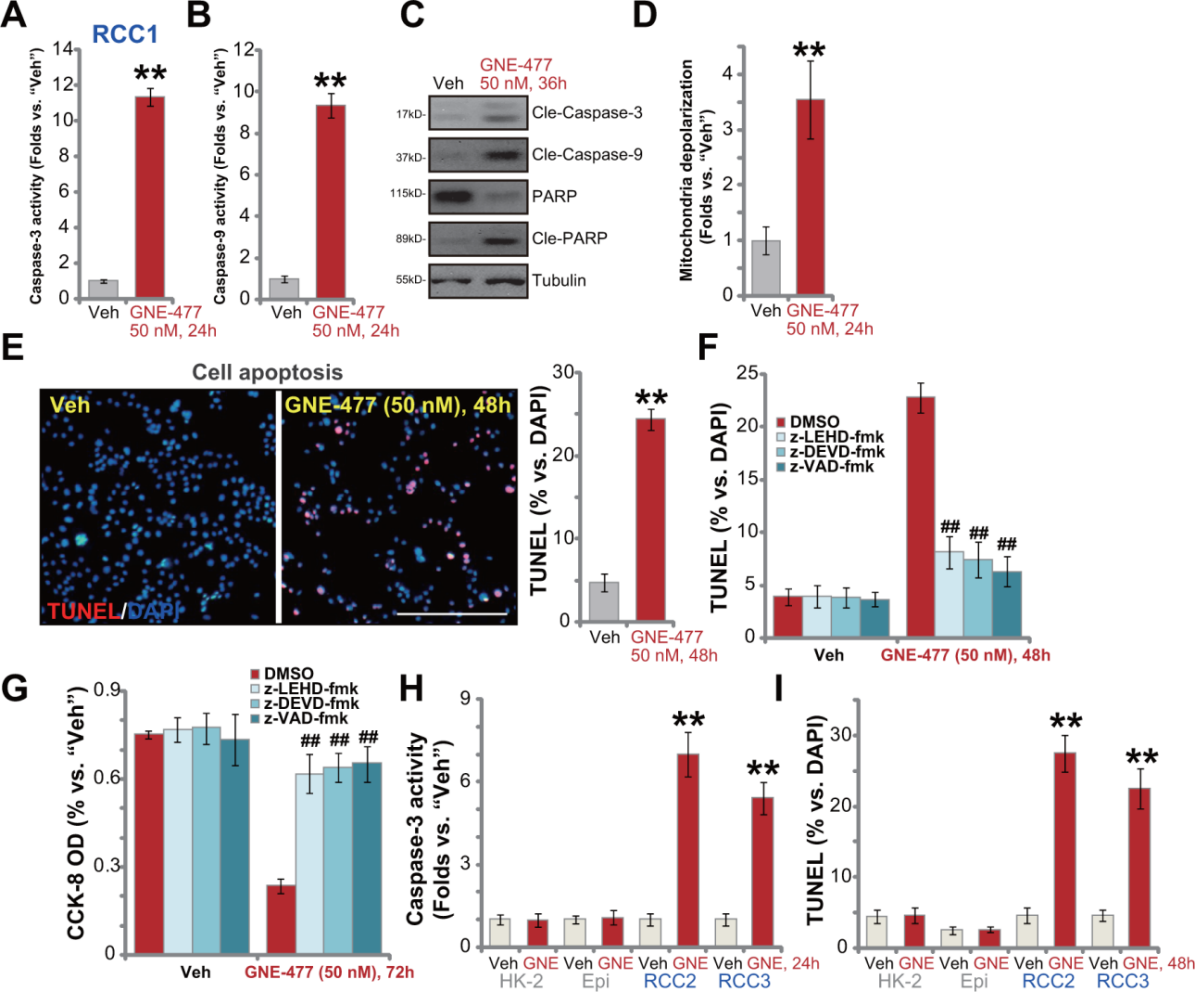
**GNE-477 induces apoptosis activation in primary human RCC cells.** The primary human RCC cells (“RCC1/RCC2/RCC3”), HK-2 renal epithelial cells (“HK-2”) or the primary human renal epithelial cells (“Epi”) were treated with GNE-477 (50 nM) or the vehicle control (“Veh”, 0.1% DMSO), cells were further cultured for designated time periods (24-48h), and cell apoptosis tested by the mentioned assays (**A**–**E**, **H**, **I**). Alternatively, RCC1 cells were pretreated for 1h with applied caspase inhibitors (each at 50 μM), followed by GNE-477 (50 nM) stimulation, cells were further cultured for 48-72h, with cell apoptosis and viability examined by nuclear TUNEL staining (**F**) and CCK-8 (**G**) assays, respectively. Bars stand for mean ± standard deviation (S.D.). For each assay, n=5. ** *p* < 0.01 *vs.* “Veh” cells (**A**, **B**, **D**, **E**, **H**, **I**). ^##^ *p* < 0.01 *vs.* “DMSO”-pretreated cells (**F**, **G**). Experiments in this figure were repeated five times, and similar results obtained. Scale bar= 200 μm (**E**).

To test cell apoptosis in GNE-477-treated RCC cells, the caspase activities were examined. As demonstrated, following GNE-477 (50 nM, 36h) treatment in RCC1 cells the caspase-3 activity (Figure 2A) and the caspase-9 activity (Figure 2B) increased over 8-10 folds (*vs.* vehicle control treatment). Western blotting assay results, Figure 2C, demonstrated that the dual PI3K-mTOR inhibitor induced cleavages of caspase-3, caspase-9 and PARP (poly (ADP-ribose) polymerase) in RCC1 cells. Further studies show that mitochondria depolarization was detected in GNE-477-treated RCC1 cells, evidenced by an increase of JC-1 green fluorescence intensity (Figure 2D). Additionally, following GNE-477 treatment about 25% of all RCC1 cell nuclei were positive for TUNEL staining (Figure 2E), indicating apoptosis activation.

To confirm that apoptosis is the primary cause of GNE-477-induced cytotoxicity in RCC1 cells, a set of different caspase inhibitors were utilized. As demonstrated, pretreatment with the caspase-3 inhibitor z-DEVD-fmk, the caspase-9 inhibitor z-LEHD-fmk, or the pan-caspase inhibitor z-VAD-fmk, potently ameliorated GNE-477-induced apoptosis activation (TUNEL assay) in RCC1 cells (Figure 2F). Consequently, GNE-477-induced cytotoxicity, evidenced by CCK-8 viability reduction, was largely inhibited (Figure 2G). In other primary human RCC cells (RCC2 and RCC3), treatment with GNE-477 (50 nM) led to robust caspase-3 activation (Figure 2H) and nuclear TUNEL ratio increase (Figure 2I), indicating apoptosis activation. On the contrary, the dual PI3K-mTOR inhibitor failed to provoke significant apoptosis in HK-2 cells and primary kidney epithelial cells (Figure 2H, 2I). Together, these results show that GNE-477 induced robust apoptosis activation in primary human RCC cells.

### GNE-477 blocks PI3K-Akt-mTOR cascade activation in primary human RCC cells

**Figure 3:**
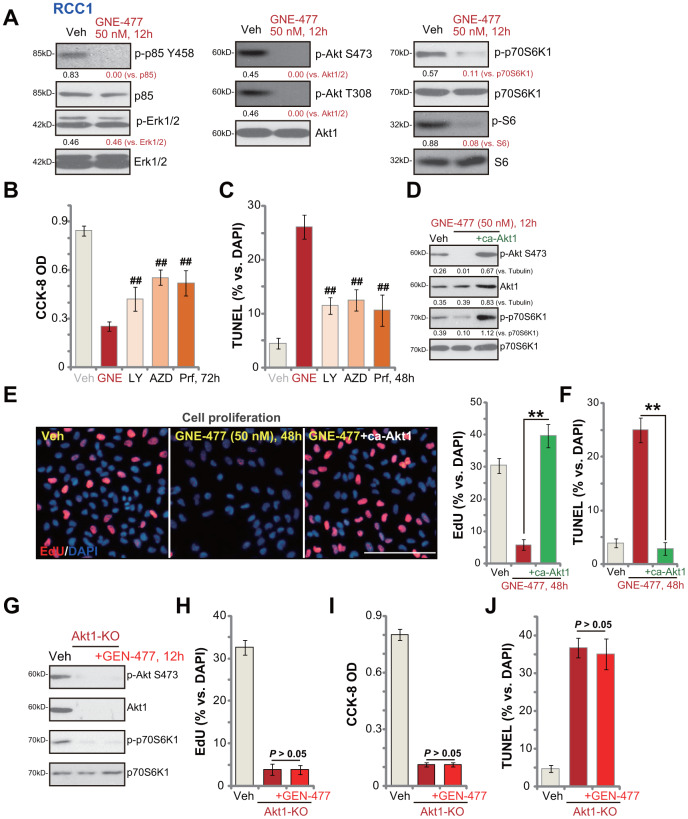
**GNE-477 blocks PI3K-Akt-mTOR cascade activation in primary human RCC cells.** RCC1 cells were treated with GNE-477 (50 nM) or the vehicle control (“Veh”, 0.1% DMSO), cells were further cultured for 12h, and expression of listed proteins tested by Western blotting (**A**); RCC1 cells were treated with GNE-477 (“GNE”, 50 nM), LY294002 (100 nM), AZD2014 (“AZD”, 100 nM), perifosine (“Prf”, 1 μM) or the vehicle control (“Veh”, 0.1% DMSO) for 48-72h, with cell viability and apoptosis tested by CCK-8 (**B**) and nuclear TUNEL staining (**C**) assays, respectively. The monoclonal stable RCC1 cells with or without the constitutively-active Akt1 (“+ca-Akt1”) construct were treated with GNE-477 (50 nM) or the vehicle control, cells were further cultured for applied time periods, expression of the listed proteins was tested (**D**); Cell proliferation and apoptosis were tested by EdU staining (**E**) and TUNEL assay (**F**), respectively. The monoclonal stable RCC1 cells with the CRISPR/Cas9 Akt1-KO construct (Akt1-KO cells) were treated with or without GNE-477 (50 nM), control cells with empty vector were treated with vehicle control (“Veh”), expression of listed proteins was shown (**G**); Cell viability, proliferation and apoptosis were tested by CCK-8 (**H**), EdU incorporation (**I**), and TUNEL staining (**J**) assays after 48h, respectively. Expression of listed proteins was quantified, normalized to the loading control (**A**, **D**). Bars stand for mean ± standard deviation (S.D.). For each assay, n=5. ^##^ *p* < 0.01 *vs.* GNE-477 treatment (**B**, **C**). ** *p* < 0.01 (**E**, **F**). Experiments in this figure were repeated five times, and similar results obtained. Scale bar= 100 μm (**E**).

Next, the potential effect of GNE-477 on PI3K-Akt-mTOR signaling cascade was tested. As shown, in primary RCC1 cells treatment with GNE-477 (50 nM, 12h) almost completely blocked phosphorylation of p85 and Akt (Ser473 and Thr308) (Figure 3A). Phosphorylation of p70S6K1 and S6, the indicator of mTORC1 activation [15, 16], was largely inhibited in GNE-477-treated RCC1 cells as well (Figure 3A). Expression of total p85, Akt1, p70S6K1 and S6 was unchanged following GNE-477 treatment (Figure 3A). Furthermore, Erk1/2 phosphorylation and total Erk1/2 expression were not altered by GNE-477 in RCC1 cells (Figure 3A). These results indicated that GNE-477 blocked the whole PI3K-Akt-mTOR cascade activation in RCC1 cells.

We next compared the anti-RCC cell activity of GNE-477 with other known PI3K-Akt-mTOR inhibitors, including a PI3K inhibitor LY294002 [17], an Akt specific inhibitor perifosine [18] and a mTOR kinase inhibitor AZD2014 [19]. In RCC1 cells, GNE-477-induced viability reduction and apoptosis (TUNEL ratio) were significantly more potent than those by LY294002, perifosine and AZD2014 (at even higher concentrations, Figure 3B, 3C).

Further studies demonstrated that a constitutively-active Akt1 (ca-Akt1) restored Akt and p70S6K1 phosphorylation in GNE-477-treated RCC1 cells (Figure 3D). Importantly, GNE-477-induced proliferation inhibition (EdU ratio decrease, Figure 3E) and cell apoptosis activation (nuclear TUNEL ratio increase, Figure 3F) were completely reversed by caAkt1 (Figure 3E, 3F). Therefore, forced activation of Akt reversed GNE-477-induced cytotoxicity in RCC1 cells, suggesting that PI3K-Akt-mTOR inhibition should be the cause of GNE-477-induced cytotoxicity against RCC cells. To further support our hypothesis, the CRISPR/Cas9 strategy was applied to complete knockout (KO) Akt1 in RCC1 cells (see Methods). Akt1 KO, mimicking GNE-477-induced activity, blocked Akt and p70S6K1 phosphorylation (Figure 3G), and led to robust viability reduction (Figure 3H), proliferation inhibition (Figure 3I) and cell apoptosis activation (Figure 3J). Importantly, in Akt1-KO RCC1 cells, adding GNE-477 (50 nM, 48h) was unable to induce further cytotoxicity (Figure 3H, 3I). These results further supported that GNE-477-induced cytotoxicity in RCC cells was due to PI3K-Akt-mTOR blockage.

### GNE-477 potently inhibits RCC xenograft tumor growth in mice

**Figure 4:**
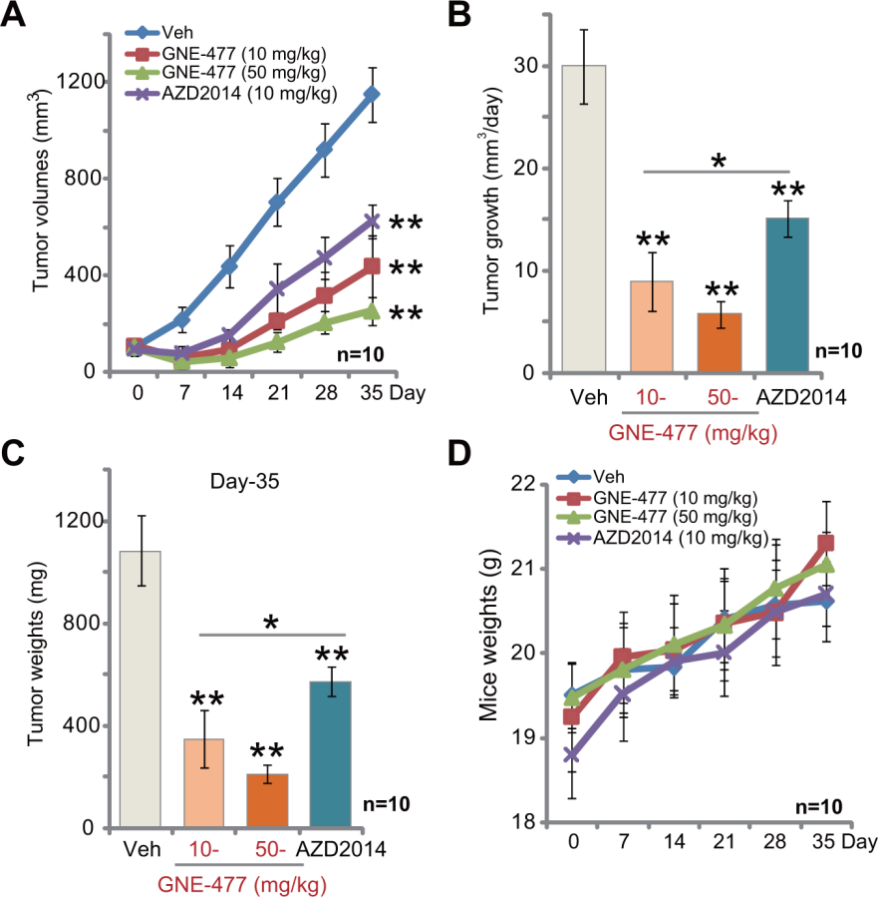
**GNE-477 potently inhibits RCC xenograft tumor growth in mice.** The nude mice bearing RCC1 xenograft tumors were intraperitoneally (*i.p.*) injected with GNE-477 (10-50 mg/kg body weight, daily, for 21 days), AZD2014 (10 mg/kg body weight, daily, for 21 days) or the vehicle control (“Veh”), tumor volumes (**A**) and mice body weights (**D**) were recorded every seven (7) days for a total of 35 days; Estimated daily tumor growth, in mm^3^ per day, was calculated (**B**); At the last day of recording (Day-35), the xenograft tumors were isolated and weighted (**C**). For each group, n=10 (mice). ** *p* < 0.01 vs. “Veh”-treated tumors (**A**–**C**). * *p* < 0.05 (**A**–**C**).

The potential anti-RCC activity of GNE-477 *in vivo* was tested. Using a previously described animal model [8, 9], we subcutaneously (*s.c.*) injected RCC1 cells to the flanks of the nude mice. Within two weeks RCC1 xenograft tumors were established with the volume close to 100 mm^3^. Tumor growth curve results, Figure 4A, demonstrated that intraperitoneal (*i.p.*) injection of GNE-477, at 10 or 50 mg/kg (daily, for 3 weeks), potently inhibited RCC1 xenograft tumor growth in nude mice. Calculating the estimated daily tumor growth, using the formula (tumor volume at Day-35—tumor volume at Day-0)/35, we again show that GNE-477 injection potently suppressed RCC1 xenograft tumor growth *in vivo* (Figure 4B). At Day-35, tumors of all three groups were isolated and weighted. Tumors of GNE-477-treated mice were significantly lighter than those of the vehicle control mice (Figure 4C).

Notably, at the dose of 10 mg/kg (*i.p.* daily for three weeks) the known mTOR kinase inhibitor AZD2014 [19–21] significantly inhibited RCC1 xenograft tumor growth in mice (Figure 4A–4C). Importantly, GNE-477 was more potent in suppressing RCC1 xenograft growth than the same concentration of AZD2014 (Figure 4A–4C). There was no significant difference in animal body weights among the four groups (Figure 4D). Furthermore, no apparent toxicities were noticed in GNE-477-treated mice and AZD2014-treated mice. These results confirmed that GNE-477, at well-tolerated doses, potently inhibited RCC1 xenograft tumor growth in mice.

## DISCUSSION

mTOR protein lies in a central position in the PI3K-Akt-mTOR cascade [15, 22, 23]. There are at least two mTORC complexes identified thus far, mTORC1 and mTORC2 [15, 22, 23]. mTORC1 is composed of mTOR, Raptor, mLST8 and several others, required for p70S6K1 and 4EBP1 phosphorylation [15, 22, 23]. mTORC2 is composed of mTOR, Rictor, Sin1, and others, serving as the upstream kinase of Akt phosphorylation at Ser-473 [24, 25]. Studies have shown that mTORC1 and mTORC2 are both essential for the progression of RCC. Both are important for cancer cell growth, proliferation and migration, angiogenesis, chemo-resistance and metastasis [10, 11, 26, 27]. Importantly, the mTOR pharmacological inhibitors have displayed therapeutic values for the treatment of RCC [10, 11, 26, 27].

mTORC1 inhibitors have been approved by the US FDA for the clinical treatment of advanced RCC patients after failure of either sunitinib or sorafenib [10, 11]. Yet, mTORC1 inhibitors could still have several limitations and drawbacks, including incomplete mTOR inhibition and feedback activation of other oncogenic signalings [10, 11].

In the present study, we show that GNE-477 blocked phosphorylation of p70S6K1-S6 and Akt (Ser-473 and Thr-308) in RCC1 cells. It thus inactivated both mTORC1 and mTORC2 cascades. Furthermore, phosphorylation of p85 was largely inhibited by GNE-477. These results show that GNE-477 blocked the whole PI3K-Akt-mTOR cascade in the RCC cells. This should explain the extremely high efficiency of this compound against RCC cells. Indeed, GNE-477 was significantly more potent than other PI3K-Akt-mTOR inhibitors (LY294002, AZD2014, perifosine) in inhibiting RCC cell survival and inducing cell apoptosis. *In vivo,* GNE-477 was more potent in suppressing RCC1 xenograft growth than AZD2014. These results support that this compound could have important therapeutic value for the treatment of RCC.

Our results imply that GNE-477-induced anti-RCC activity is due to PI3K-Akt-mTOR inhibition. Restoring Akt-mTOR activation, by a ca-Akt1 construct, completely reversed GNE-477-induced cytotoxicity against RCC1 cells. Furthermore, Akt1 depletion, by the CRISPR/Cas9 Akt1 KO construct, mimicked GNE-477’s activity and potently inhibited RCC1 cell viability and proliferation. Importantly, adding GNE-477 in the Akt1-KO RCC1 cells failed to induce further cytotoxicity. These results suggest that PI3K-Akt-mTOR inactivation by GNE-477 led to cytotoxicity and growth inhibition in RCC cells. This should also explain why GNE-477 was completely ineffective in HK-2 cells and primary human kidney epithelial cells. Since previous studies have shown that the basal PI3K-Akt-mTOR activation is quite low in the normal epithelial cells [28–30]. We also show that GNE-477 did not induce apparent toxicities to the nude mice.

## CONCLUSIONS

Together, we conclude that targeting PI3K-Akt-mTOR by GNE-477 inhibited human RCC cell growth *in vitro* and *in vivo*. It should be noted that a number of tested PI3K-mTOR kinase inhibitors failed to result in significant clinical improvement for RCC patients [10, 11]. Certain PI3K-mTOR inhibitors are even more toxic and less efficacious than everolimus or temsirolimus [31]. Therefore, the current results of *in vitro* and animal studies could not be directly translated to humans, and thus the efficacy and safety of GNE-477 will definitely need further characterizations and research. Lower concentrations of GNE-477 could also be tested in mice.

## MATERIALS AND METHODS

### Chemicals and reagents

GNE-477 was synthesized by Min-de Biotech Co. (Suzhou, China) based on the published procedure [14]. LY294002, perifosine and AZD2014 were purchased from Selleck (Beijing, China). The caspase-3 inhibitor z-DEVD-fmk, the caspase-9 inhibitor z-LEHD-fmk, and the pan-caspase inhibitor z-VAD-fmk were obtained from RiboBio (Guangzhou, China). Antibodies of this study were obtained from Cell Signaling Tech (Beverly, MA) and Abcam Co. (Suzhou, China). All cell culture regents were from Gibco Co. (Shanghai, China). Puromycin, polybrene and JC-1 dye were provided by Sigma (St. Louis, MO).

### Cell culture

As described previously [8, 9] the primary human RCC cells (from Dr. Zheng at Nantong University [32]) were derived from three independent primary RCC patients with written-informed consents. They were named as “RCC1”, “RCC2” and “RCC3”. The primary cancer cells were cultured in the previously-described medium for primary cells [33]. Cultures of HK-2 cells and primary human renal epithelial cells were also described in our previous study [9]. Protocols of this study were approved by the Ethics Board of Wenzhou Medical University, according to the principles expressed in the Declaration of Helsinki.

### Cell viability

The primary human RCC cells or the epithelial cells were seeded onto 96-well plates (at 4, 000 cells per well). After the indicated treatments, the cell viability was examined by a Cell Counting Kit-8 (CCK-8) (Dojindo Molecular Technologies, Japan) CCK-8 optical densities (ODs) were examined using a microplate reader at the test-wavelength of 550 nm.

### EdU (5-ethynyl-20-deoxyuridine) incorporation

The primary human RCC cells or the epithelial cells were seeded onto the six-well tissue-culturing plates (1×10^5^ cells per well). Following the indicated treatments an EdU Apollo-567 assay kit (RiboBio, Guangzhou, China) was utilized to test cell proliferation, with nuclear EdU and DAPI staining visualized under a fluorescent microscope (1×200 magnification, Leica, Shanghai, China.). In each treatment five random views with total 500 cells were included to calculate the nuclear EdU ratio (% *vs.* DAPI).

### *In vitro* cell migration and invasion

The primary human RCC cells or the epithelial cells, with the applied treatments, were initially seeded on the upper chambers of “Transwell” (BD Biosciences, Shanghai, China) [34], at a density of 1×10^4^ cells in 250 μL serum-free medium in each chamber. The complete medium (15% FBS) was added to the lower chambers. After incubation for 24h, on the lower surface the migrated cells were stained and counted manually. To test cell invasion, Matrigel (Sigma, Shanghai, China) was added on the upper chambers of “Transwell”. For each condition, five repeated views were included to calculate the average number of migrated/invaded cells.

### Cell cycle assay

The primary human RCC cells with or without GNC-477 treatment were stained with propidium iodide (PI, 5 μg/mL, Thermo-Fisher Invitrogen, Shanghai, China) and RNase (50 μg/mL, Thermo-Fisher Invitrogen). DNA contents were examined under a flow cytometer (BD Biosciences, Franklin Lakes, NJ). Cell cycle distributions were recorded.

### Colony formation

RCC1 cells, in 0.25% agarose (Sigma)-containing complete medium, were initially seeded onto 10-cm tissue-culture dishes (at 1×10^4^ cells per dish). The FBS-containing complete medium with or without GNE-477 (at tested concentrations) was renewed every 2 days (for a total of 10 days). Afterwards, viable cell colonies were counted manually.

### TUNEL (terminal deoxynucleotidyl transferase dUTP nick end labeling) assay

The protocols of nuclear TUNEL staining were described previously [8, 35]. Following the applied GNE-477 treatment, RCC cells were co-stained with TUNEL and DAPI. The TUNEL ratio (% *vs.* DAPI), calculating 500 nuclei per treatment in five random views (1:200), was recorded.

### Western blotting

As described previously [8, 9], following the applied treatment, the quantified protein lysates (30-40 μg per sample) were separated by SDS-PAGE gels, that were transferred to the PVDF (polyvinylidene difluoride) blots (Merck-Millipore, Shanghai, China). After blocking (in milk-containing PBST), the blots were incubated with applied primary and secondary antibodies. The protein bands were visualized based on the molecular weights by using an enhanced chemiluminescence (ECL) kit (Pierce) [36]. Data quantification was through an ImageJ software (NIH, US).

### Mitochondrial depolarization

As described [37], with mitochondrial depolarization in the stressed cells JC-1 red fluorescein shall aggregate into mitochondria to form green monomers [38]. Briefly, after the indicated treatments, RCC1 cells were stained with JC-1 dye at 10 μg/mL for 30 min under the dark. The JC-1 green intensity was examined via a fluorescence spectrofluorometer at 550 nm.

### Constitutively-active mutant Akt1

The recombinant constitutively-active Akt1-GFP (caAkt1, S473D) adenovirus was provided by Dr. Zhang [39], that was transduced to RCC1 cells (cultured in the polybrene-containing complete medium). Cells were than subjected to FACS sorting of GFP to establish the monoclonal stable RCC1 cells, with caAkt1 expression verified by Western blotting analyses.

### Akt1 knockout

A lenti-CRISPR/Cas9-GFP Akt1-KO construct was from Dr. Zhang’s lab at Soochow University [40], transduced to primary RCC1 cells. Cells were then subjected to FACS to sort GFP-positive cells, which were further distributed to 96-well tissue culture plates, with Akt1 knockout (KO) screened. The monoclonal stable Akt1 KO RCC1 cells were established.

### Mice xenografts

As described previously [9], the female nude mice (5-6 week of age, 18.2-19.1 grams in weights) were provided by the Animal Center of Wenzhou Medical University (Wenzhou, China), maintained under standard conditions. Six million RCC1 cells per mouse were inoculated *s.c.* to the right flanks. Within two weeks the xenograft tumors were established with the volume close to 0.1 cm^3^. The tumor-bearing mice were randomly assigned into three groups (n=10 per group), intraperitoneally injected with GNE-477 or the vehicle control [14]. Tumor recordings were described early [8, 9]. The protocols were approved by the IACUC of Wenzhou Medical University, according to National Institutes of Health guide for the care and use of laboratory animals.

### Statistical analyses

Data were presented as mean ± standard deviation (SD). Statistics were analyzed by one-way ANOVA using a SPSS software (21.0, SPSS Co., Chicago, CA). To test difference between two specific groups, a two tailed T Test was applied (Excel 2007, Microsoft). *P* values <0.05 were considered statistically different.

## References

[1] Motzer RJ, Hutson TE, Cella D, Reeves J, Hawkins R, Guo J, Nathan P, Staehler M, de Souza P, Merchan JR, Boleti E, Fife K, Jin J, et al. Pazopanib versus sunitinib in metastatic renal-cell carcinoma. N Engl J Med. 2013; 369:722–31. 10.1056/NEJMoa1303989 23964934

[2] Cohen HT, McGovern FJ. Renal-cell carcinoma. N Engl J Med. 2005; 353:2477–90. 10.1056/NEJMra043172 16339096

[3] Motzer RJ, Bander NH, Nanus DM. Renal-cell carcinoma. N Engl J Med. 1996; 335:865–75. 10.1056/NEJM199609193351207 8778606

[4] Siegel R, Ma J, Zou Z, Jemal A. Cancer statistics, 2014. CA Cancer J Clin. 2014; 64:9–29. 10.3322/caac.21208 24399786

[5] Siegel RL, Miller KD, Jemal A. Cancer Statistics, 2017. CA Cancer J Clin. 2017; 67:7–30. 10.3322/caac.21387 28055103

[6] Siegel RL, Miller KD, Jemal A. Cancer statistics, 2016. CA Cancer J Clin. 2016; 66:7–30. 10.3322/caac.21332 26742998

[7] Wettersten HI, Weiss RH. Potential biofluid markers and treatment targets for renal cell carcinoma. Nat Rev Urol. 2013; 10:336–44. 10.1038/nrurol.2013.52 23545813

[8] Ye X, Xie J, Huang H, Deng Z. Knockdown of MAGEA6 Activates AMP-Activated Protein Kinase (AMPK) Signaling to Inhibit Human Renal Cell Carcinoma Cells. Cell Physiol Biochem. 2018; 45:1205–18. 10.1159/000487452 29448247

[9] Ye XT, Huang H, Huang WP, Hu WL. LncRNA THOR promotes human renal cell carcinoma cell growth. Biochem Biophys Res Commun. 2018; 501:661–67. 10.1016/j.bbrc.2018.05.040 29752937

[10] Pal SK, Quinn DI. Differentiating mTOR inhibitors in renal cell carcinoma. Cancer Treat Rev. 2013; 39:709–19. 10.1016/j.ctrv.2012.12.015 23433636 PMC4957946

[11] Husseinzadeh HD, Garcia JA. Therapeutic rationale for mTOR inhibition in advanced renal cell carcinoma. Curr Clin Pharmacol. 2011; 6:214–21. 10.2174/157488411797189433 21827395

[12] Konings IR, Verweij J, Wiemer EA, Sleijfer S. The applicability of mTOR inhibition in solid tumors. Curr Cancer Drug Targets. 2009; 9:439–50. 10.2174/156800909788166556 19442061

[13] Motzer RJ, Escudier B, Oudard S, Hutson TE, Porta C, Bracarda S, Grünwald V, Thompson JA, Figlin RA, Hollaender N, Urbanowitz G, Berg WJ, Kay A, et al, and RECORD-1 Study Group. Efficacy of everolimus in advanced renal cell carcinoma: a double-blind, randomised, placebo-controlled phase III trial. Lancet. 2008; 372:449–56. 10.1016/S0140-6736(08)61039-9 18653228

[14] Heffron TP, Berry M, Castanedo G, Chang C, Chuckowree I, Dotson J, Folkes A, Gunzner J, Lesnick JD, Lewis C, Mathieu S, Nonomiya J, Olivero A, et al. Identification of GNE-477, a potent and efficacious dual PI3K/mTOR inhibitor. Bioorg Med Chem Lett. 2010; 20:2408–11. 10.1016/j.bmcl.2010.03.046 20346656

[15] Saxton RA, Sabatini DM. mTOR Signaling in Growth, Metabolism, and Disease. Cell. 2017; 168:960–76. 10.1016/j.cell.2017.02.004 28283069 PMC5394987

[16] Sabatini DM. mTOR and cancer: insights into a complex relationship. Nat Rev Cancer. 2006; 6:729–34. 10.1038/nrc1974 16915295

[17] Brunn GJ, Williams J, Sabers C, Wiederrecht G, Lawrence JC Jr, Abraham RT. Direct inhibition of the signaling functions of the mammalian target of rapamycin by the phosphoinositide 3-kinase inhibitors, wortmannin and LY294002. EMBO J. 1996; 15:5256–67. 10.1002/j.1460-2075.1996.tb00911.x 8895571 PMC452270

[18] Kondapaka SB, Singh SS, Dasmahapatra GP, Sausville EA, Roy KK. Perifosine, a novel alkylphospholipid, inhibits protein kinase B activation. Mol Cancer Ther. 2003; 2:1093–103. 14617782

[19] Pike KG, Malagu K, Hummersone MG, Menear KA, Duggan HM, Gomez S, Martin NM, Ruston L, Pass SL, Pass M. Optimization of potent and selective dual mTORC1 and mTORC2 inhibitors: the discovery of AZD8055 and AZD2014. Bioorg Med Chem Lett. 2013; 23:1212–16. 10.1016/j.bmcl.2013.01.019 23375793

[20] Zheng B, Mao JH, Qian L, Zhu H, Gu DH, Pan XD, Yi F, Ji DM. Pre-clinical evaluation of AZD-2014, a novel mTORC1/2 dual inhibitor, against renal cell carcinoma. Cancer Lett. 2015; 357:468–75. 10.1016/j.canlet.2014.11.012 25444920

[21] Huo HZ, Zhou ZY, Wang B, Qin J, Liu WY, Gu Y. Dramatic suppression of colorectal cancer cell growth by the dual mTORC1 and mTORC2 inhibitor AZD-2014. Biochem Biophys Res Commun. 2014; 443:406–12. 10.1016/j.bbrc.2013.11.099 24309100

[22] Laplante M, Sabatini DM. mTOR signaling in growth control and disease. Cell. 2012; 149:274–93. 10.1016/j.cell.2012.03.017 22500797 PMC3331679

[23] Lamming DW, Ye L, Sabatini DM, Baur JA. Rapalogs and mTOR inhibitors as anti-aging therapeutics. J Clin Invest. 2013; 123:980–89. 10.1172/JCI64099 23454761 PMC3582126

[24] Sarbassov DD, Ali SM, Kim DH, Guertin DA, Latek RR, Erdjument-Bromage H, Tempst P, Sabatini DM. Rictor, a novel binding partner of mTOR, defines a rapamycin-insensitive and raptor-independent pathway that regulates the cytoskeleton. Curr Biol. 2004; 14:1296–302. 10.1016/j.cub.2004.06.054 15268862

[25] Sarbassov DD, Guertin DA, Ali SM, Sabatini DM. Phosphorylation and regulation of Akt/PKB by the rictor-mTOR complex. Science. 2005; 307:1098–101. 10.1126/science.1106148 15718470

[26] Mihaly Z, Sztupinszki Z, Surowiak P, Gyorffy B. A comprehensive overview of targeted therapy in metastatic renal cell carcinoma. Curr Cancer Drug Targets. 2012; 12:857–72. 10.2174/156800912802429265 22515521 PMC3434473

[27] Barthélémy P, Hoch B, Chevreau C, Joly F, Laguerre B, Lokiec F, Duclos B. mTOR inhibitors in advanced renal cell carcinomas: from biology to clinical practice. Crit Rev Oncol Hematol. 2013; 88:42–56. 10.1016/j.critrevonc.2013.02.006 23523056

[28] Cho DC, Cohen MB, Panka DJ, Collins M, Ghebremichael M, Atkins MB, Signoretti S, Mier JW. The efficacy of the novel dual PI3-kinase/mTOR inhibitor NVP-BEZ235 compared with rapamycin in renal cell carcinoma. Clin Cancer Res. 2010; 16:3628–38. 10.1158/1078-0432.CCR-09-3022 20606035 PMC2905505

[29] Pan XD, Gu DH, Mao JH, Zhu H, Chen X, Zheng B, Shan Y. Concurrent inhibition of mTORC1 and mTORC2 by WYE-687 inhibits renal cell carcinoma cell growth in vitro and in vivo. PLoS One. 2017; 12:e0172555. 10.1371/journal.pone.0172555 28257457 PMC5336203

[30] Zhu H, Mao JH, Wang Y, Gu DH, Pan XD, Shan Y, Zheng B. Dual inhibition of BRD4 and PI3K-AKT by SF2523 suppresses human renal cell carcinoma cell growth. Oncotarget. 2017; 8:98471–81. 10.18632/oncotarget.21432 29228703 PMC5716743

[31] Powles T, Lackner MR, Oudard S, Escudier B, Ralph C, Brown JE, Hawkins RE, Castellano D, Rini BI, Staehler MD, Ravaud A, Lin W, O’Keeffe B, et al. Randomized Open-Label Phase II Trial of Apitolisib (GDC-0980), a Novel Inhibitor of the PI3K/Mammalian Target of Rapamycin Pathway, Versus Everolimus in Patients With Metastatic Renal Cell Carcinoma. J Clin Oncol. 2016; 34:1660–68. 10.1200/JCO.2015.64.8808 26951309 PMC5569691

[32] Gu DH, Mao JH, Pan XD, Zhu H, Chen X, Zheng B, Shan Y. microRNA-302c-3p inhibits renal cell carcinoma cell proliferation by targeting Grb2-associated binding 2 (Gab2). Oncotarget. 2017; 8:26334–43. 10.18632/oncotarget.15463 28412750 PMC5432261

[33] Zheng B, Mao JH, Li XQ, Qian L, Zhu H, Gu DH, Pan XD. Over-expression of DNA-PKcs in renal cell carcinoma regulates mTORC2 activation, HIF-2α expression and cell proliferation. Sci Rep. 2016; 6:29415. 10.1038/srep29415 27412013 PMC4944168

[34] Sun J, Huang W, Yang SF, Zhang XP, Yu Q, Zhang ZQ, Yao J, Li KR, Jiang Q, Cao C. Gαi1 and Gαi3mediate VEGF-induced VEGFR2 endocytosis, signaling and angiogenesis. Theranostics. 2018; 8:4695–709. 10.7150/thno.26203 30279732 PMC6160771

[35] Xie J, Li Q, Ding X, Gao Y. GSK1059615 kills head and neck squamous cell carcinoma cells possibly via activating mitochondrial programmed necrosis pathway. Oncotarget. 2017; 8:50814–23. 10.18632/oncotarget.15135 28881606 PMC5584207

[36] Zhou LN, Li P, Cai S, Li G, Liu F. Ninjurin2 overexpression promotes glioma cell growth. Aging (Albany NY). 2019; 11:11136–47. 10.18632/aging.102515 31794427 PMC6932907

[37] Wang Y, Liu J, Tao Z, Wu P, Cheng W, Du Y, Zhou N, Ge Y, Yang Z. Exogenous HGF Prevents Cardiomyocytes from Apoptosis after Hypoxia via Up-Regulating Cell Autophagy. Cell Physiol Biochem. 2016; 38:2401–13. 10.1159/000445592 27299574

[38] Brooks MM, Neelam S, Fudala R, Gryczynski I, Cammarata PR. Lenticular mitoprotection. Part A: monitoring mitochondrial depolarization with JC-1 and artifactual fluorescence by the glycogen synthase kinase-3β inhibitor, SB216763. Mol Vis. 2013; 19:1406–12. 23825920 PMC3695757

[39] Zhang D, Xia H, Zhang W, Fang B. The anti-ovarian cancer activity by WYE-132, a mTORC1/2 dual inhibitor. Tumour Biol. 2016; 37:1327–36. 10.1007/s13277-015-3922-0 26293898

[40] Zhu JL, Wu YY, Wu D, Luo WF, Zhang ZQ, Liu CF. SC79, a novel Akt activator, protects dopaminergic neuronal cells from MPP^+^ and rotenone. Mol Cell Biochem. 2019; 461:81–89. 10.1007/s11010-019-03592-x 31342299

